# Label-free stimulated Raman scattering microscopy visualizes changes in intracellular morphology during human epidermal keratinocyte differentiation

**DOI:** 10.1038/s41598-019-49035-x

**Published:** 2019-08-29

**Authors:** Mariko Egawa, Shinya Iwanaga, Junichi Hosoi, Makiko Goto, Haruyo Yamanishi, Masashi Miyai, Chika Katagiri, Kyoya Tokunaga, Takuya Asai, Yasuyuki Ozeki

**Affiliations:** 1 Shiseido Global Innovation Center, Yokohama, 220–0011 Japan; 2 Department of Electrical Engineering and Information Systems, Graduate School of Engineering, The University of Tokyo, Tokyo, 113–8656 Japan

**Keywords:** Cell biology, Imaging and sensing, Organelles, Raman spectroscopy

## Abstract

Epidermal keratinocyte (KC) differentiation, which involves the process from proliferation to cell death for shedding the outermost layer of skin, is crucial for the barrier function of skin. Therefore, in dermatology, it is important to elucidate the epidermal KC differentiation process to evaluate the symptom level of diseases and skin conditions. Previous dermatological studies used staining or labelling techniques for this purpose, but they have technological limitations for revealing the entire process of epidermal KC differentiation, especially when applied to humans. Here, we demonstrate label-free visualization of three-dimensional (3D) intracellular morphological changes of *ex vivo* human epidermis during epidermal KC differentiation using stimulated Raman scattering (SRS) microscopy. Specifically, we observed changes in nuclei during the initial enucleation process in which the nucleus is digested prior to flattening. Furthermore, we found holes left behind by improperly digested nuclei in the stratum corneum, suggesting abnormal differentiation. Our findings indicate the great potential of SRS microscopy for discrimination of the degree of epidermal KC differentiation.

## Introduction

Skin is the largest organ in the body and has various functions that are essential to maintaining life activities, such as forming a barrier against dry environments (by preventing water evaporation) and harmful external substances. Generally, skin is classified into three layers: (from outermost to innermost) the epidermis, dermis, and subcutaneous tissue^1^. Among these layers, the epidermis is most important for skin barrier function. The epidermis consists of four distinct sublayers: (from outer to inner) the stratum corneum (SC), stratum granulosum (SG), stratum spinosum (SS), and stratum basale (SB). Keratinocytes (KCs) undergo morphological changes as they progress from the SB to SC during epidermal differentiation. Specifically, each KC must rid itself of its nucleus, flatten, die, and fill with keratin fibres during epidermal terminal differentiation in the SG to SC^2^. These steps result in the generation of a healthy SC with protective barrier function. The process of epidermal maturation to generate a healthy SC is especially important to provide our bodies with a strong protective barrier^3^. Atopic dermatitis and psoriasis are typical disorders related to the differentiation process. Skin conditions associated with abnormal differentiation are also observed in non-diseased skin, like the condition of parakeratosis, whereby the nucleus remains in the SC. Skin roughness has been observed as a result of less severe abnormalities, sometimes observed in dry environment conditions^3^. Thus, elucidation of the epidermal KC differentiation process is crucial in dermatology to evaluate the symptom level of diseases involving epidermal KC differentiation as well as skin conditions such as disorders related to epidermal metabolism.

Until recently, many dermatological studies using fixation/staining or labelling with fluorescent dyes or proteins at the genetic level have had limited success in revealing epidermal maturation by focusing on natural moisturizing factors^4^, barrier function^5^, intercellular lipids^6^, desquamation processes, and the molecular mechanism of epidermal terminal differentiation^7,8^. However, such methods have been known to affect the main components of the epidermis: water, lipids, and proteins. In addition, observing staining of the SC remains difficult using these biological methods because it is dyed non-selectively. Therefore, observation of cell morphologies during the epidermal KC differentiation process is an important target that, until now, was not previously available. Recent studies using transgenic labelling revealed shapes of KCs in the SG, namely that apical paracellular spaces of KCs were sealed with tight junctions and the basic shape of tight junction-bearing cells was a flattened Kelvin’s tetrakaidecahedron^9–11^. These studies employed combinations of labelling techniques and microscopy; however, such techniques cannot be performed on humans because they rely on the use of labelling applied as a fluorescent dye in advance or by inducing genetic-level changes to proteins *in vivo*. Thus, epidermal KC differentiation has not been fully elucidated in humans using these previously reported biological techniques.

Recent progress in optical imaging techniques such as confocal microscopy^12^, multiphoton microscopy^13^, and optical coherence tomography^14^ has enabled the non-invasive visualization and structural analysis of skin layers. These non-invasive optical techniques have previously been applied to *in vivo* measurements of the human epidermis^15–17^. However, it remains difficult to visualize epidermal KC differentiation using these non-invasive optical methods. Therefore, we focused on the application of stimulated Raman scattering (SRS) microscopy^18–20^ to evaluate the epidermal KC differentiation process at a cellular level without staining/labelling. SRS microscopy enables faster imaging of various functional groups of molecules^21–29^ compared with conventional spontaneous Raman scattering microscopy^30,31^. For dermatological applications, SRS microscopy was employed to visualize drug delivery to the skin by taking advantage of high measurement speeds^32–38^. We previously reported our preliminary label-free observations of epidermal cells in both cell culture and tissue using SRS microscopy. Our results indicated the potential applications of SRS microscopy to the dermatological investigation of cell lineages and types by cellular-level analysis^39^. However, elucidation of epidermal KC differentiation has not been fully accomplished in humans even using SRS microscopy.

Thus, the purpose of the current study was to visualize human epidermal KC differentiation without fixing/staining/labelling using SRS microscopy to clarify currently unknown intracellular morphological changes. We examined 19 abdominal and eyelid skin without any chemical pretreatment to visualize morphological changes in epidermal KC differentiation, especially targeting cell flattening, enucleation, and keratinization. We have previously presented our preliminary results^40^. We have continued these investigations and report the results in this paper.

## Results

### *Ex vivo* visualization of intracellular morphologies of the epidermis

**Figure 1:**
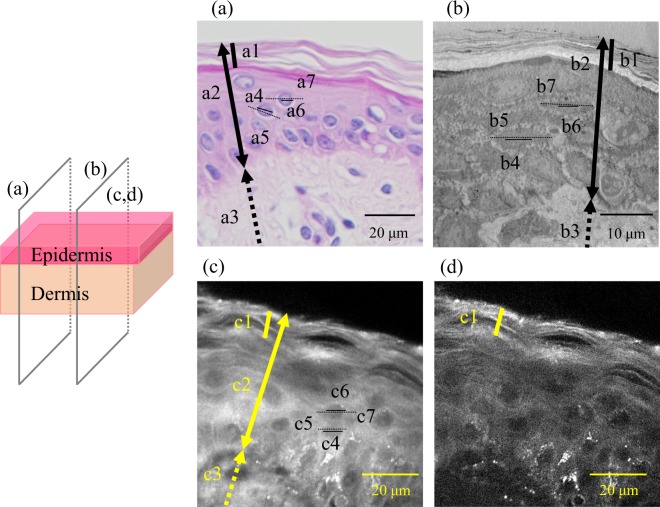
Comparison of stimulated Raman scattering (SRS) microscopy with conventional dermatological methods. (**a**) Haematoxylin-eosin staining, (**b**) transmission electron microscopy, (**c**) 2930 cm^−1^ SRS microscopy image mainly reflecting proteins, and (**d**) 2850 cm^−1^ SRS microscopy image mainly reflecting lipids. All images show vertical sections of abdominal skin of a 64-year-old Caucasian female. The same vertical section images for transmission electron microscopy and SRS were observed. Thick lines in a1, b1, and c1 indicate the stratum corneum. Thick lines with double arrows in a2, b2, and c2 indicate the full epidermis. Thick dashed lines with single arrows in a3, b3, and c3 indicate part of the dermis. Lines in a4, a6, b4, b6, c4, and c6 indicate the long diameter of nuclei. Dashed lines in a5, a7, b5, b7, c5, and c7 indicate the long diameter of keratinocytes.

Vertical cross-section images of abdominal skin of a 64-year-old subject observed by haematoxylin-eosin (HE) staining, transmission electron microscopy (TEM), SRS imaging at 2930 cm^−1^, which mainly reflects proteins, and 2850 cm^−1^ images that mainly reflect lipids, are shown in Fig. 1. As shown in the illustration in Fig. 1, the same vertical section images for TEM and SRS. The optical vertical cross-section image observed by SRS was similar to the two conventional dermatological techniques with regard to the morphology of each epidermal layer. However, subtle differences such as thicknesses of the SC [Fig. 1(a1, b1, c1)], size of cell [Fig. 1(a5, a7, b5, b7, c5, c7)], and size of nuclei [Fig. 1(a4, a6, b4, b6, c4, c6)] were observed. Specifically, the thickness of the SC and full epidermis by HE staining, TEM, and SRS were 9.9, 6.0, 9.3 and 50.1, 31.2, 48.1 µm, respectively. The average long diameters of cells and nuclei by HE staining, TEM, and SRS were 13.9, 10.9, 12.0 and 3.7, 3.0, 4.6 µm, respectively. The difference in values was suggested to be the effect of chemical pretreatment processes such as fixing/labelling/staining used in conventional dermatological techniques on the obtained images of tissue staining.

**Figure 2:**
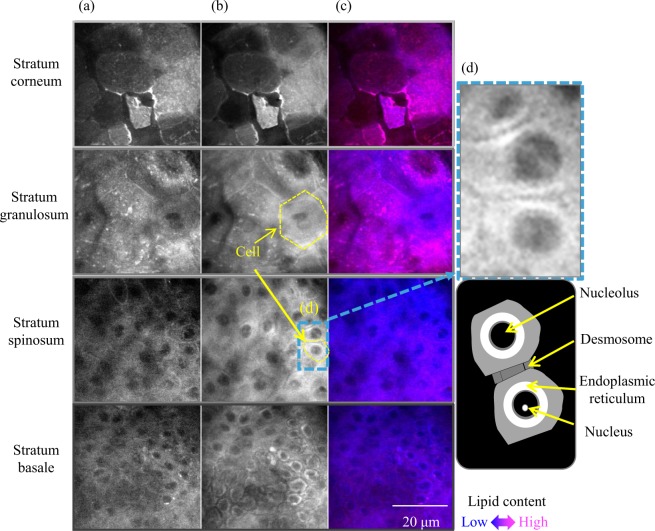
Typical molecular vibrational images in horizontal cross-sections of each epidermal layer of abdominal skin of a 70-year-old subject. (**a**) Images at 2850 cm^−1^ mainly reflect lipids, while those at (**b**) 2930 cm^−1^ mainly reflect proteins. Brightness correction was performed in each image for this figure. Merged images (**c**) were created with non-brightness-corrected images to calculate the ratio of 2850 cm^−1^ and 2930 cm^−1^ intensities by pseudocolour with 2850 cm^−1^, red and 2930 cm^−1^, blue.

Figure 2 shows typical molecular vibrational images of each epidermal layer in optical horizontal unsliced cross-sections of skin from a 70-year-old Caucasian female. Characteristic intracellular morphologies of each epidermal layer were observed without labelling, similar to the previously reported preliminary results of a 52-year-old Caucasian female at various depths^39^. In the SB and SS, nuclei were observed as a low intensity area both at 2850 cm^−1^, reflecting mainly lipids (Fig. 2b), and at 2930 cm^−1^, reflecting mainly proteins (Fig. 2a); whereas, nucleoli were observed as a high intensity area in 2930 cm^−1^ images (Fig. 2d). Lipid-rich granules in the SG analogized as lamellar granules in which lipids accumulated in the SG. A relatively high intensity at 2850 cm^−1^ was observed as a red colour in the SC in the merged image (Fig. 2c), thus reflecting the existence of intercellular lipids. In addition, visualization of the SC without distinct layers was observed in some skin (as for this subject), a feature not observed in the previously reported 52-year-old subject^39^.

### 3D intracellular morphologies of skin

Supplementary videos S1 and S2 show typical horizontal cross-section images at 2930 cm^−1^, reflecting mainly proteins, of abdominal skin of a 56-year-old subject and eyelid skin of a 59-year-old subject respectively. The 3D images consist of optical horizontal cross-sections at a 1-µm interval in depth. The differentiation process was clearly observed in 3D, as differences in intracellular morphology between the two tissues were clearly observed in each epidermal layer; for example, differences in the flattened features of the outlined KCs in the SB and SS, intranuclear features during the enucleation process in the upper part of SS and SG, and roughness in the outline of dead KCs in the SC.

### Changes in the size of KCs during epidermal differentiation

**Figure 3:**
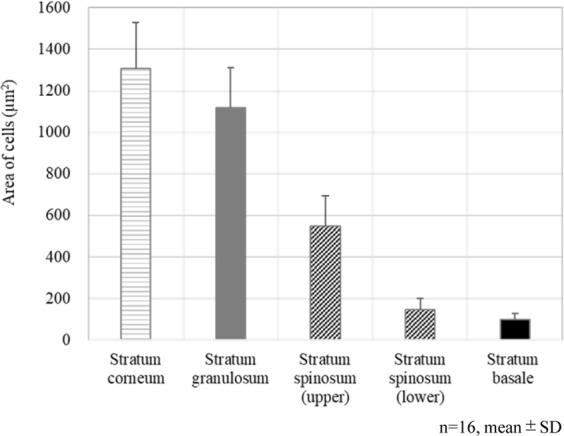
Changes in the size of keratinocytes during epidermal keratinocyte differentiation. Average area of each layer in 16 subjects.

Continuous cell size changes accompanying epidermal KC differentiation from SB to the SC were measured. Sizes of KCs in each layer were calculated from three values on one horizontal cross section image on the SB, SS, SG, and the lower/middle layers of the SS and two values on the upper layer of the SC where three could not be calculated, then the average of each layer was calculated. Changes in KC size during epidermal differentiation are shown in Fig. 3. The average cell size at the SB (14 abdominal skin of 31, 31, 33, 39, 43, 49, 49, 52, 56, 60, 62, 66, 70, and 74 years old subjects; and two eyelid skin of 59 and 60 years old subjects) was approximately 99.5 µm^2^, which increased up to 1120 µm^2^ at the SG, and finally reached 1305 µm^2^ at the SC. The size of cells increased more than 10 times at the SC compared with the SB during differentiation.

### Intercellular morphological changes of nuclei in the SS

**Figure 4:**
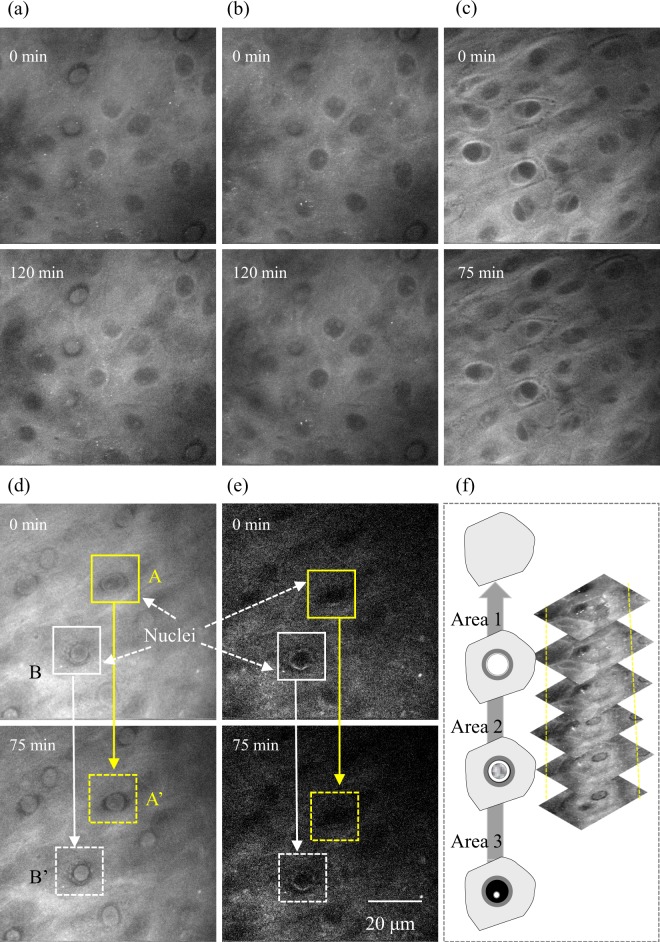
Typical time-lapse optical horizontal cross-section images in the stratum spinosum in abdominal skin of 39 and 49-year-old subjects. (**a**) 2930 cm^−1^ of skin of a 39-year-old subject (corresponding to Areas 1 and 2), (**b**) 1 µm lower part of (**a**) (corresponding to Areas 2 and 3), (**c**) 2930 cm^−1^ of skin of a 49-year-old subject (corresponding to Area 3), (**d**) 2930 cm^−1^, and (**e**) 2850 cm^−1^ of skin of a 49-year-old subject (corresponding to Areas 1 and 2). Time-lapse images were obtained without any treatment of the tissues. Regions A, B to A’, B’ show time-dependent changes in intracellular and nuclear morphology. The diagram in (**f**) shows suggested changes in nuclei during the enucleation process and optical cross-section images at 2930 cm^−1^ at a near depth of (**d**).

Figure 4 shows typical time-lapse optical cross-section images at 2850 cm^−1^ and 2930 cm^−1^ in the SS of skin of 39- and 49-year-old subjects from an abdominal site. Horizontal cross-section SRS images of skin mounted onto a cover slip with PBS were obtained as a measurement of 0 min, then in the absence of any chemical treatment the same area was measured using the same method after 75 or 120 min. The diagram in Fig. 4f shows suggested changes in the nuclei during the enucleation process and optical cross-section images at 2930 cm^−1^. As already shown in Fig. 2a, nuclei in KCs in the middle to lower part of the SS were observed as a black low-intensity area, while nucleoli appeared as a white high-intensity area in living layers in the 2930 cm^−1^ image, reflecting mainly proteins. As shown in Fig. 4a–c, similar images were obtained in skin of a 39-year-old subject (corresponding to Areas 1 and 2) and in skin of a 49-year-old subject (corresponding to Area 3) at 0 min and after 75 or 120 min. Of note, filling with protein was observed in areas A and B of Fig. 4d,e in 2930 cm^−1^ images, but not in the 2850 cm^−1^ image in the skin of a 49-year-old subject (corresponding to Areas 1 and 2).

### Existence of holes in the SC

**Figure 5:**
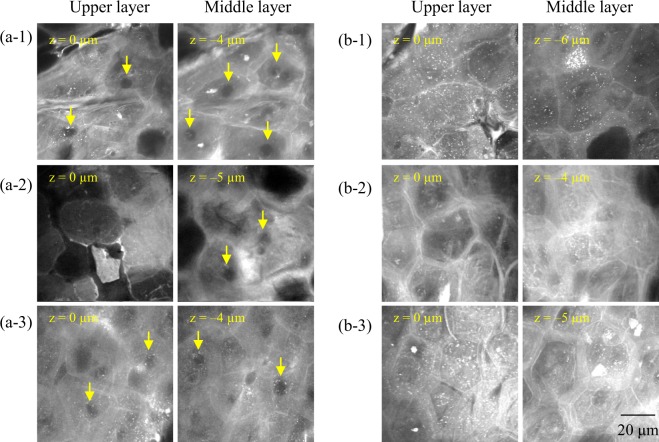
Holes in the stratum corneum. Optical cross-section images at 2930 cm^−1^ mainly reflect proteins in the stratum corneum. Yellow arrows indicate some of the holes. (**a**1–3) Images of abdominal sites of 33-, 70-, and 74-year-old Caucasian females, respectively, suggest that keratin embedding was not completed after enucleation. (**b**1–3) Images of abdominal skin sites of 39-, 49-, and 56-year-old Caucasian females, respectively, showing no holes.

**Figure 6:**
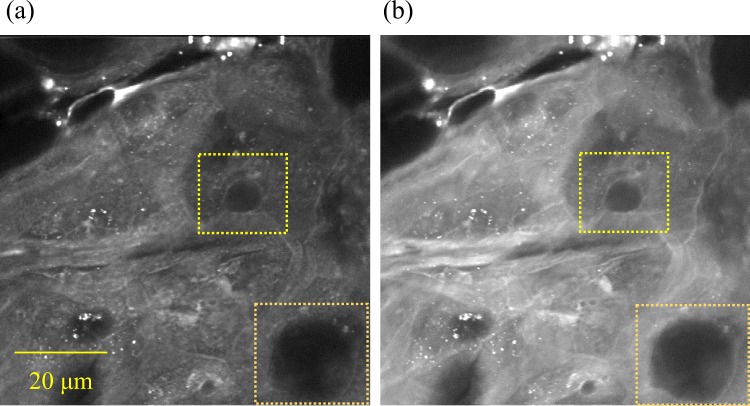
Molecular vibrational characteristics of holes in the stratum corneum. Optical cross-section images of an abdominal site of a 33-year-old Caucasian female at (**a**) 2850 cm^−1^ and (**b**) 2930 cm^−1^ are shown.

We observed holes in the SC in horizontal cross-section images, whereby the intensity at both 2850 cm^−1^ (reflecting mainly lipids) and 2930 cm^−1^ (reflecting mainly proteins) was quite low, almost black. We named these “SC holes”. Figure 5 shows typical SC holes in 2930 cm^−1^ images. Some SC holes are highlighted with yellow arrows. We observed SC holes in certain skin samples [Fig. 5(a1–a3)], but some samples did not exhibit SC holes [Fig. 5(b1–b3)]. Figure 6 shows the molecular vibrational characteristics of SC holes. Based on the observations of 2850 cm^−1^ (Fig. 6a) and 2930 cm^−1^ (Fig. 6b) images, the outline of SC holes consisted of lipid-rich components.

### Ambiguous cell outlines in the SC as a result of aging

**Figure 7:**
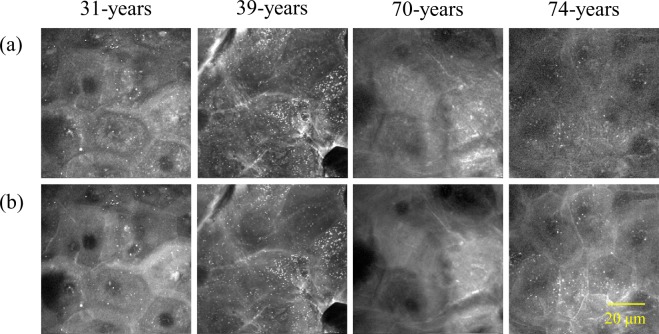
Ambiguous cell outlines in the stratum corneum. Optical cross-section images at (**a**) 2850 cm^−1^ and (**b**) 2930 cm^−1^ of different-aged skin.

Figure 7 shows typical horizontal cross-section images of the SC at 2850 cm^−1^ (reflecting mainly lipids) and 2930 cm^−1^ (reflecting mainly proteins) in skin of different ages. Cell outlines were unclear in aged skin but were clear in skin from younger individuals. Differences in the vulnerability of the cell membrane or abnormally formed intercellular lipids are potential reasons underlying this observation.

## Discussion

In the current study, we were able to achieve the first 3D constructions visualizing changes in intracellular morphology during epidermal KC differentiation in humans without any chemical processing that might affect the skin. These results demonstrated the great potential of intracellular morphology imaging with molecular vibration information using SRS microscopy for quick diagnosis of the differentiation state of epidermal layers. Our approach was different from most other studies using spontaneous/coherent anti-Stokes Raman microscopy, which relies on the use of characteristic molecular vibrations of target components^30,31,41^. Our vibrational contrast approach takes advantage of the benefits of high-speed evaluation to yield medical imaging applications.

By analysing changes in the morphology of KCs during epidermal differentiation, we evaluated intact cell size from SB to SC for the first time. Evaluating the size of cells at the skin surface is an alternative to measuring epidermal turnover using a stripped SC^42^. Our method enabled us to evaluate actual turnover conditions by observing the size of cells in each epidermal cell layer. Thus, it will be possible to elucidate mechanisms underlying turnover in humans *in vivo* using non-labelling molecular imaging with back-scattering detection.

In the upper part of the SS to SG, we observed the shrinking of nuclei and subsequent filling with protein in the upper part of the SS during the initial stage of the enucleation process. Time-dependent changes in spectral images allowed visualization of the contraction process of cell nuclei. In these observations, the inside of nuclei were exhibited as a high-intensity area in the 2930 cm^−1^ image from the outline of nuclei to the inside, followed by subsequent protein filling of nuclei, which occurred at 75 min (Fig. 4d), resulting because of nuclear shrinkage at the initial stage of the enucleation process or changes associated with tissue deterioration in refrigeration storage. In either case, this is the first example of visualizing such intracellular morphological changes *ex vivo*. In previous studies conducted by a labelling method using a 3D skin model and mice, change in morphology of nuclei in the last moment of the enucleation process was captured on a time scale within 60 minutes^43,44^. The time scale of morphological changes in the nucleus we captured do not seem to contradict these previous studies. In addition, our observation of tight junction-like features in the SG of humans (Supplementary videos S1 and S2) was similar to those reported using the transgenic labelling of mice^9^. Our findings demonstrating the visualization of human tight junctions using vibrational contrast illustrate the potential of SRS for applications in dermatological basic research.

In the SC, we obtained lipid-rich images based on high levels of intercellular lipids. Samples taken from younger subjects showed higher lipid levels that made the cell boundaries clearly visible. However, lipid levels were lower in elderly skin, yielding an ambiguous cell outline, suggesting that advancing age causes a deterioration of the epidermal KC differentiation process. We also observed holes, circular regions with low protein densities, in the SCs of some subjects. Notably, parakeratosis, a condition in which nuclei remain in the SC, was not observed in any skin by HE-staining (Supplementary Fig. S1). A part of this phenomenon, referred to as the “ghost nucleus”, was captured by a staining method in a previous report using tape-stripped SC cells at the outermost thin layer of skin^41^. However, it was impossible to clarify their identity in detail because of the limitations of staining/chemical treatment methods especially for the SC. In the process of healthy SC formation, the nuclei of KCs disappear in the SG, the inside of the KCs— including the lost part of the nucleus— is filled with keratin fibres in the SG to lower part of the SC, and the cell shape becomes flattened and finally a sheet form stacked as ten to several tens of layers is formed^1,45^. This layered structure of the SC possesses a strong barrier function. Barrier function is decreased during parakeratosis, which is in the category of non-diseased skin, because the SC is not completely filled with keratin fibres because the nuclei remain. Our observations suggest that the nuclear membrane would remain intact, such that keratin fibres did not fill the inside remnant of the nuclear membrane, thus leaving a distinct hole after digestion of the nucleus. Incomplete SC with holes remaining would lead to a decrease in physical barrier function. Such holes appearing would be the result of abnormalities in the enucleation process, especially the process of nuclear membrane loss and keratin fibre filling after enucleation. Importantly, the barrier function of skin with SC holes would be lower than that of normal skin, resulting in the absorption and retention of water in the SC, which would be affected. These are the first findings implying the order of digestion in the enucleation process. Thus, morphological changes during epidermal KC terminal differentiation from the upper part of the SS to the SG are considered important factors for cell death in the SC. Abnormalities in the steady process of cell death in the SC, as discussed above, might lead to both a decline in the barrier function of the epidermis and reduced moisture retention.

As for the obtainable information, SRS microscopy still has some limitations compared with existing technologies. First, image resolution for evaluation of the intracellular morphology of SRS microscopy is not high compared with TEM. Second, functional groups detectable by SRS microscopy within a narrow range of wavenumber measurements are limited compared with spontaneous Raman scattering microscopy and coherent anti-Stokes Raman scattering microscopy. However, the biggest benefit of SRS microscopy is fast-speed imaging. Compared with other high-speed morphology imaging methods available for the epidermis, such as confocal microscopy^12^ and optical coherence tomography^14^, SRS microscopy has the advantage of visualizing the morphology of intracellular components using vibrational contrast of video-rate images. Future investigation with back-scattering detection without slicing^18,21,32^ would greatly expand the potential applications of SRS microscopy for evaluating skin diseases in clinical dermatology, as well as skin conditions in cosmetic dermatology fields. Furthermore, discrimination of the differentiation degree by intracellular morphology with vibrational contrast could be applied beyond dermatology for the evaluation of stem cells and cell differentiation in a variety of fields.

## Conclusions

This is the first report demonstrating changes in 3D intracellular morphology during epidermal KC differentiation *ex vivo* using subcellular spatial distribution without fixing/staining/labelling. We analysed actual turnover conditions by observing the size of KCs in each epidermal layer, which was not possible using previous dermatological/optical techniques. In addition, we observed changes in intracellular and nuclear morphology during the enucleation stage for the first time, which allowed us to determine that the digestion of nuclei after enucleation is an important step linked to terminal differentiation in the SC. We also revealed the existence of holes in the SC that we posit are related to stiffness and water absorption/release levels. Our results can be applied to clarify which differentiation processes are important in forming healthy skin with a strong barrier function. We also showed that high-speed SRS imaging has great potential even in a narrow wavenumber range to evaluate the degree of differentiation by visualizing intracellular morphology.

## Methods

### SRS microscopy

**Figure 8:**
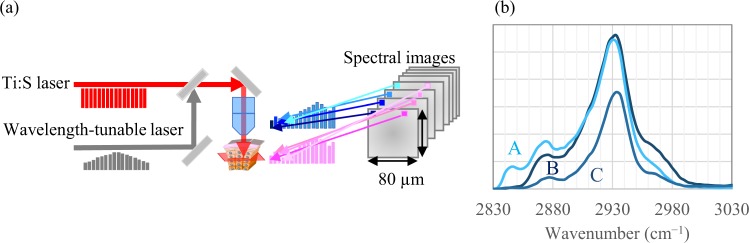
Apparatus overview of stimulated Raman scattering microscopy (**a**) and typical spectra of epidermal layers (**b**); (A) stratum corneum, (B) stratum basale, and (C) stratum spinosum.

The details have been described previously^46^. Briefly, a Ti:sapphire (Ti:S) laser at 790 nm with 0.14-nm spectrum width and wavelength-tunable Yb fibre (YbF) laser at 1015–1045 nm were used as light sources. An apparatus overview is shown in Fig. 8a. The power of Ti:S and YbF lasers at the input of laser scanners was 110–120 mW each, and that in the sample plane was estimated to be −60 mW each. Horizontal cross-section SRS images (80 × 80 μm; 500 × 500 pixels; transmission mode; horizontal spatial resolution of −0.5 μm) were acquired in the wavenumber range of 2800 to 3100 cm^−1^, corresponding to the C–H stretching region^20,21,39^. The frame rate of the microscope was 30 frames/s. Images were accumulated to improve the signal-to-noise ratio (continuous wavenumber scanning mode; 10 accumulations, discrete scanning mode; 50 or 100 accumulations). The wavelength dependence of the YbF laser intensity was measured on each measurement day and used for calibration.

### *Ex vivo* molecular imaging of human skin

Human skin from the abdomen and eyelid (n = 19, 31–74 years old, Caucasian females) were purchased from Biopredic International (Rennes, France) via KAC Co., Ltd. (Kyoto, Japan). The tissues had been collected during plastic surgery after informed consent had been obtained according to French laws including ethics regulations. Our study was approved by the ethics committee of Shiseido, in accordance with the guidelines of the National Institute of Health. Skin were dermatomed to approximately 400-μm thickness on the production date in France and then transported to Japan. Tissues were refrigerated during transit and until measurement without any chemical treatment. Skin were immersed in PBS, mounted onto a cover slip, sandwiched with another coverslip, and sealed with enamel. The tissues were molecularly imaged in the horizontal optical cross-section at a depth interval of 1 µm using SRS microscopy without physical slicing. Typical SRS spectra of epidermal layers are shown in Fig. 8b. Spectral images at 2850 cm^−1^ (CH_2_ symmetric stretching), which mainly reflect lipids, and 2930 cm^−1^ (CH_3_ symmetric stretching), which mainly reflect proteins, were used to visualize the intracellular morphology of epidermal layers^25,29,34,35,47^.

### Dermatological analysis

To compare SRS microscopy with conventional dermatological methods, HE staining and scanning electron microscopy^48^ were performed.

### Image analysis

Acquired 16-bit molecular vibrational images were analysed by ImageJ software (v1.48; National Institutes of Health, Bethesda, MD), IMARIS software (Imaris 8.4.1; Bitplane AG, Zurich, Switzerland), and in-house image analysis software.

## Supplementary information

Supplementary Information

Supplementary Video S1

Supplementary Video S2
