# Supplementary material for: Label-free stimulated Raman scattering microscopy visualizes changes in intracellular morphology during human epidermal keratinocyte differentiation
Source: Sci Rep. 2019 Aug 29;9:12601. doi: 10.1038/s41598-019-49035-x (PMC6715667; doi:10.1038/s41598-019-49035-x)
Supplement: Supplementary file 1 — Supplementary Information [file 41598_2019_49035_MOESM1_ESM.pdf]

Supplementary Information

Label-free stimulated Raman scattering microscopy visualizes changes in intracellular morphology during human epidermal keratinocyte differentiation

Mariko Egawa[1,*], Shinya Iwanaga[1], Junichi Hosoi[1], Makiko Goto[1], Haruyo Yamanishi[1], Masashi Miyai[1], Chika Katagiri[1], Kyoya Tokunaga[2], Takuya Asai[2], and Yasuyuki Ozeki[2]

[1] Shiseido Global Innovation Center, Yokohama 220–0011, Japan

[2] Department of Electrical Engineering and Information Systems, Graduate School of Engineering, The University of Tokyo, Tokyo 113–8656, Japan

Supplementary Figure S1, Supplementary Video S1, and Supplementary Video S2.

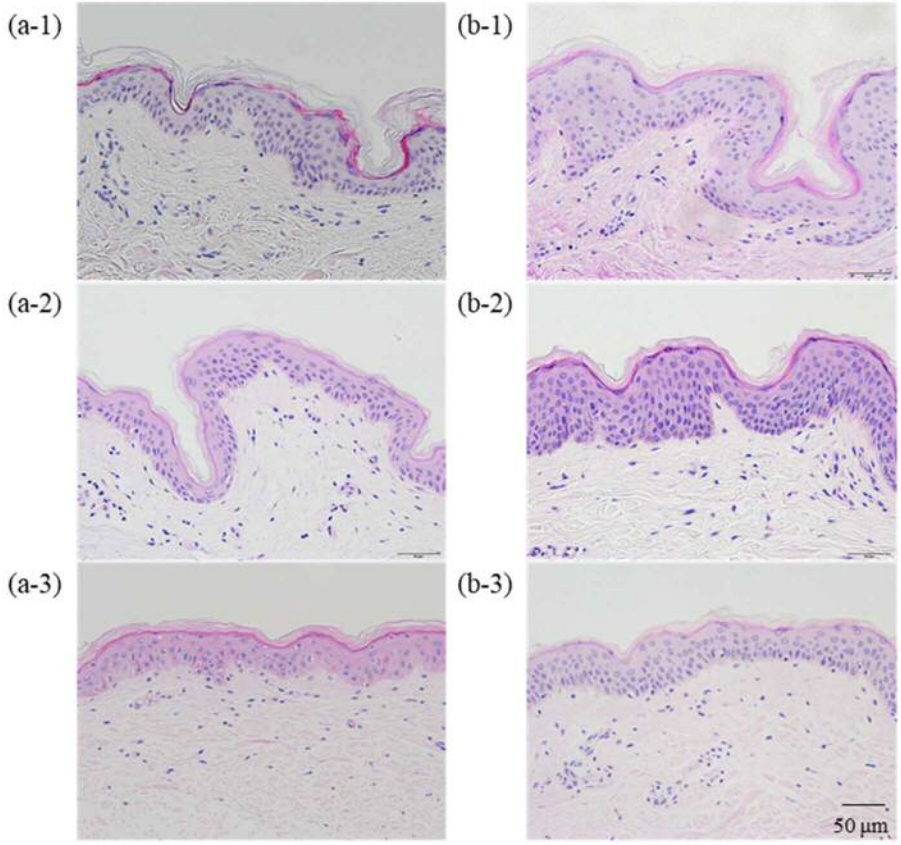

Supplementary Figure S1. Vertical cross-section images of haematoxylin-eosin staining correspond to the optical cross-section images at 2930 cm^{-1} shown in Fig. 5. (a1–3) shows staining images of abdominal skin of 33-, 70-, and 74-year-old subjects and (b1–3) shows those of 39-, 49-, and 56-year-old subjects, respectively. Parakeratosis (state where the nucleus remains in the dead cells of the stratum corneum) was not observed in any skin.

Supplementary Video S1. Three-dimensional intracellular morphologies of abdominal skin of a 56-year-old subject. The stratum corneum, stratum granulosum, stratum spinosum, and stratum basal (from outermost to innermost). The size of each optical horizontal cross-section image is 80 μm in the vertical and horizontal directions at a 1-μm interval in depth.

Supplementary Video S2. Three-dimensional intracellular morphologies of eyelid skin of a 59-year-old subject. The stratum corneum, stratum granulosum, stratum spinosum, and stratum basal (from outermost to innermost). The size of each optical horizontal cross-section image is 80 μm in the vertical and horizontal directions at a 1-μm interval in depth.
